# Impact of age on efficacy and toxicity of nilotinib in patients with chronic myeloid leukemia in chronic phase: ENEST1st subanalysis

**DOI:** 10.1007/s00432-017-2402-x

**Published:** 2017-03-31

**Authors:** Francis J. Giles, Delphine Rea, Gianantonio Rosti, Nicholas C. P. Cross, Juan Luis Steegmann, Laimonas Griskevicius, Philipp le Coutre, Daniel Coriu, Ljubomir Petrov, Gert J. Ossenkoppele, Francois-Xavier Mahon, Susanne Saussele, Andrzej Hellmann, Perttu Koskenvesa, Tim H. Brümmendorf, Gunther Gastl, Fausto Castagnetti, Beatrice Vincenzi, Jens Haenig, Andreas Hochhaus

**Affiliations:** 1 Division of Hematology Oncology, Northwestern University Feinberg School of Medicine, Chicago, IL USA; 2 Service d’Hématologie Adulte, Hôpital Saint-Louis, Paris, France; 3 Department of Experimental, Diagnostic and Specialty Medicine, “Seràgnoli” Institute of Hematology, “S. Orsola-Malpighi” University Hospital, University of Bologna, Bologna, Italy; 4 Faculty of Medicine, University of Southampton, Southampton, UK; 5 Hematology Department, IIS-IP, Hospital Universitario de la Princesa, Madrid, Spain; 6 Vilnius University Hospital Santariskiu Clinics, Vilnius University, Vilnius, Lithuania; 7 Charité – Universitätsmedizin Berlin Campus Virchow, Berlin, Germany; 8 Fundeni Clinical Institute, University of Medicine “Carol Davila” Bucharest, Bucharest, Romania; 9 Ion Chiricuta Instititute of Oncology, Cluj-Napoca, Romania; 10 Department of Hematology, VU University Medical Center, Amsterdam, The Netherlands; 11 Hematology Laboratory, Bordeaux University, Bordeaux, France; 12 Universitätsmedizin Mannheim, Universität Heidelberg, Mannheim, Germany; 13 Department of Hematology, Medical University of Gdansk, Gdansk, Poland; 14 Helsinki University Hospital Comprehensive Cancer Center, Department of Hematology and Hematology Research Unit Helsinki, University of Helsinki, Helsinki, Finland; 15 Department of Hematology, Oncology, Hemostaseology, and Stem Cell Transplantation, University Hospital of the RWTH Aachen, Aachen, Germany; 16 Internal Medicine V, Hematology and Oncology, Medical University Innsbruck, Innsbruck, Austria; 17 Novartis Oncology Italy, Origgio, VA Italy; 18 Novartis Pharma GmbH, Nuremberg, Germany; 19 Klinik für Innere Medizin II/Hämatologie, Onkologie, Universitätsklinikum Jena, Jena, Germany

**Keywords:** Chronic myeloid leukemia, Nilotinib, Frontline, Impact of age, Clinical trial, Molecular response

## Abstract

**Purpose:**

Achievement of deep molecular response with a tyrosine kinase inhibitor in patients with chronic myeloid leukemia (CML) is required to attempt discontinuation of therapy in these patients. The current subanalysis from the Evaluating Nilotinib Efficacy and Safety in Clinical Trials as First-Line Treatment (ENEST1st) study evaluated whether age has an impact on the achievement of deeper molecular responses or safety with frontline nilotinib in patients with CML.

**Methods:**

ENEST1st is an open-label, multicenter, single-arm, prospective study of nilotinib 300 mg twice daily in patients with newly diagnosed CML in chronic phase. The patients were stratified into the following 4 groups based on age: young (18–39 years), middle age (40–59 years), elderly (60–74 years), and old (≥75 years). The primary end point was the rate of molecular response 4 ([MR^4^] *BCR–ABL1* ≤0.01% on the international scale) at 18 months from the initiation of nilotinib.

**Results:**

Of the 1091 patients enrolled, 1089 were considered in the analysis, of whom, 23% (*n* = 243), 45% (*n* = 494), 27% (*n* = 300), and 5% (*n* = 52) were categorized as young, middle age, elderly, and old, respectively. At 18 months, the rates of MR^4^ were 33.9% (95% confidence interval [CI], 27.8–40.0%) in the young, 39.6% (95% CI, 35.3–44.0%) in the middle-aged, 40.5% (95% CI, 34.8–46.1%) in the elderly, and 35.4% (95% CI, 21.9–48.9%) in the old patients. Although the incidence of adverse events was slightly different, no new specific safety signals were observed across the 4 age groups.

**Conclusions:**

This subanalysis of the ENEST1st study showed that age did not have a relevant impact on the deep molecular response rates associated with nilotinib therapy in newly diagnosed patients with CML and eventually on the eligibility of the patients to attempt treatment discontinuation.

## Introduction

Old age was considered as a negative prognostic factor for the treatment of chronic myeloid leukemia (CML) and predicted poor survival outcomes with earlier treatment regimens prior to the use of tyrosine kinase inhibitors (TKIs) (Kantarjian et al. 1987; Silver et al. 1999; Berger et al. 2003). With the advent of imatinib, the overall survival (OS) of patients with CML improved substantially (Kantarjian et al. 2006; Hochhaus et al. 2009). A number of studies have evaluated the effect of age with imatinib, which suggested that imatinib was able to nullify the negative effect of age on outcomes with CML therapy (Breccia et al. 2012; Proetel et al. 2014).

Second-generation TKIs, including nilotinib and dasatinib, were developed for the treatment of patients, who were resistant to or intolerant of imatinib. Nilotinib (Tasigna^®^) was first approved for patients with CML, who were resistant to or intolerant of imatinib, and subsequently for newly diagnosed patients with CML (Tasigna 2015). The pivotal study, Evaluating Nilotinib Efficacy and Safety in Clinical Trials-Newly Diagnosed Patients (ENESTnd), showed higher rates of major molecular response (MMR; *BCR–ABL1* ≤0.1% on the International Scale [IS]) and lower rates of progression with two doses (300 and 400 mg) of nilotinib compared to imatinib (Hochhaus et al. 2016b). Recent data have shown that achievement of deeper molecular responses, molecular response 4 ([MR^4^] *BCR*–*ABL1* ^IS^ ≤0.01%) and molecular response 4.5 ([MR^4.5^] *BCR–ABL1* ^IS^ ≤0.0032%), results in better treatment outcomes in patients with CML (Falchi et al. 2013; Etienne et al. 2014; Hehlmann et al. 2014). Additionally, in studies investigating treatment-free remission (TFR) in patients with CML, the eligibility criteria are the achievement of stable deep molecular responses (Hughes et al. 2016; Saglio et al. 2016). The ENEST1st study was, therefore, conducted to evaluate the efficacy of nilotinib in achieving deeper molecular responses in a large patient population of newly diagnosed patients with CML who were *BCR–ABL1* positive (Hochhaus et al. 2016a). The results from the study showed that a majority of the patients achieved MR^4^ by 24 months with progression-free survival (PFS) rate of almost 100%.

Studies evaluating the impact of age on the safety and efficacy of second-generation TKIs, including nilotinib, are limited. The limited data available on the effect of age only evaluated the response rates for broader categories like elderly patients with age greater than 60 or 65 and younger patients (le Coutre et al. 2009; Larson et al. 2011). The current subanalysis of the ENEST1st study evaluated the impact of age on the deep molecular response and safety with frontline nilotinib in four major categories of patients based on age.

## Methods

### Patients

The subanalysis included all patients enrolled in the multicenter ENEST1st study, which recruited patients from 26 European countries. The inclusion and exclusion criteria have been previously published (Hochhaus et al. 2016a). Briefly, male or female patients with Philadelphia chromosome (Ph) or *BCR–ABL1* + CML in chronic phase (CML-CP), aged ≥18 years and were within 6 months of diagnosis of the disease, were enrolled. Patients were also required to have a World Health Organization performance status of ≤2. Patients who had prior treatment with hydroxyurea (>6 months) or imatinib (>3 months) were not included. Patients with ventricular-paced pacemaker, congenital long QT syndrome, QTcF >450 ms, myocardial infarction within the past 12 months, or other clinically significant heart disease were excluded. In addition, patients with impaired gastrointestinal function, concurrent uncontrolled medical conditions that would present unacceptable safety risks or compromise compliance with the protocol, or concomitant treatment with medications with the potential to prolong the QT interval or known to be strong inducers or inhibitors of cytochrome P450 3A4 are also excluded. Informed consent was obtained from each patient in writing before any study-specific procedures were performed.

### Study design and treatment

The ENEST1st was a multicenter, single-arm study evaluating nilotinib at 300 mg twice daily (bid) in newly diagnosed and previously untreated patients. ENEST1st was registered in the EU Clinical Trials Registry (2009-017775-19) and ClinicalTrials.gov (NCT01061177). In this subanalysis, the patients were stratified to four subgroups according to age at the time of study entry. The 4 groups that were defined for the purpose of this study were young patients (18–39 years), middle-aged patients (40–59 years), elderly patients (60–74 years), and old patients (≥75 years). The primary end point was the rate of MR^4^ at 18 months from the initiation of nilotinib. The secondary end points included rates of MR^4^ and MR^4.5^ at various time points, OS, and PFS. Safety was evaluated throughout the study, and National Cancer Institute (NCI) Common Terminology Criteria for Adverse Events (CTCAE) version 4.0 for toxicity and adverse event (AE) reporting was used to report AEs. All patients were treated with an initial dose of nilotinib 300 mg bid for up to 24 months. Dose escalation was not allowed beyond 300 mg bid of nilotinib. Dose reductions were permitted for grade 3 or 4 hematologic AEs related to white blood cells or platelets and for clinically significant nonhematological AEs of severity greater than grade 2. The study protocol for the ENEST1st study did not require regular monitoring of lipid and glucose profiles.

The study protocol was approved by the independent ethics committee (IEC) or institutional review board (IRB) for each center and was conducted according to the ethical principles of the Declaration of Helsinki.

### Assessments

The molecular response rates assessed by the *BCR–ABL1* transcript levels determined by multiplex polymerase chain reaction (PCR) at baseline and subsequently every 3 months by real-time quantitative PCR at a designated European Treatment Outcome Study (EUTOS) laboratory standardized to IS. Samples with a total of <10,000 *ABL1* transcripts or <32,000 *ABL1* transcripts were considered as not evaluable for MR^4^ or MR^4.5^, respectively. In the study, PFS was defined as the time from start of the study drug to the earliest progression to accelerated phase or blast crisis (AP/BC) or death from any cause, and OS was defined as the time from the start of the study drug to death from any cause.

### Statistical analyses

The intention-to-treat (ITT) population and the safety sets consisted of all patients who received at least one dose of study drug and was used for demographics, baseline characteristics, efficacy analyses, and safety. For the evaluation of the molecular response, only those patients in the ITT population with typical *BCR–ABL1* transcripts at screening, i.e., b3a2 and/or b2a2, were considered. For calculation of response rates “at” a designated time point, patients were considered responders only if an assessment at that time point showed achievement of the response. Response rates “by” a designated time point were calculated as cumulative response rates, counting all patients with a response detected at or before the specified time point as responders. All response rates were calculated as raw proportions. Rates of freedom from progression to AP/BC on treatment and OS were estimated using Kaplan–Meier product limit estimates according to ITT principles.

## Results

### Patients

**Fig. 1:**
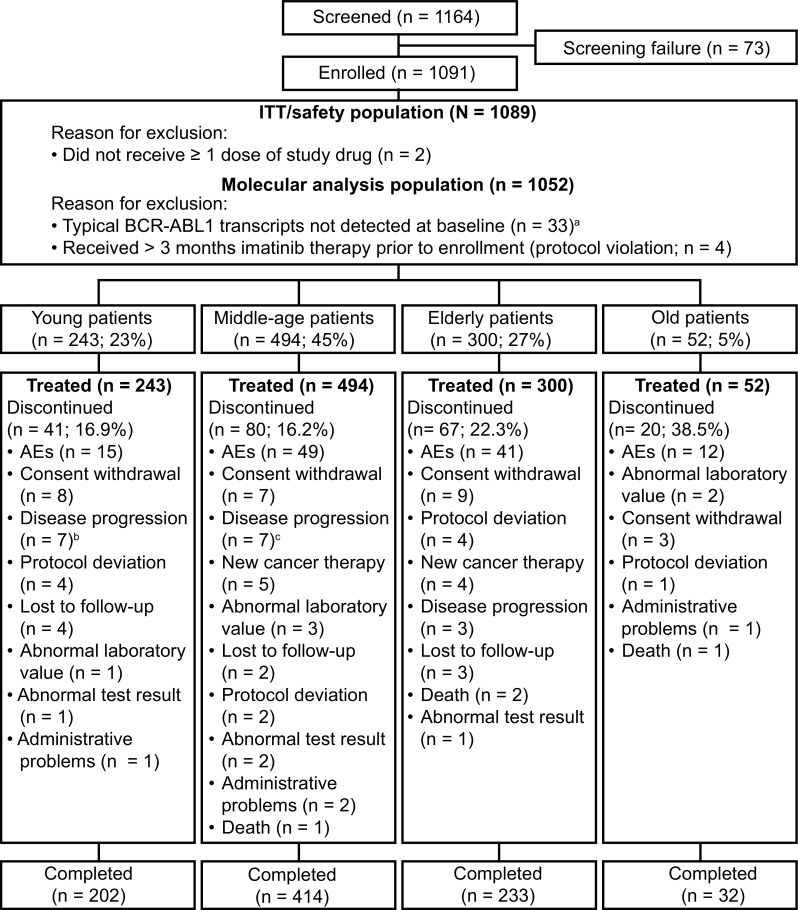
Patient disposition. ^a^Patients not in the molecular analysis population were distributed in the age groups as follows: 9 patients in young, 18–39 years old, 10 patients in middle-aged, 40–59 years old, 10 patients in elderly, 60–74 years old, 4 patients in old, ≥75 years old, groups; ^b^two patients in the young group who discontinued due to progression to AP/BC are considered under disease progression; ^c^four patients in the middle-aged group who discontinued due to progression to AP/BC are considered under disease progression. *ITT* intention–to-treat, *Ph* philadelphia chromosome

In the ENEST1st study, from 2010 to 2012, 1164 patients were screened and 1091 patients were enrolled across 26 European countries in 307 sites (Fig. 1). Of the 1091 patients, 1089 patients who received ≥1 dose of nilotinib 300 mg bid were considered in the ITT analysis. Of the 1089 patients, 23% (*n* = 243), 45% (*n* = 494), 27% (*n* = 300), and 5% (*n* = 52) were categorized as young, middle age, elderly, and old, respectively, according to the defined criteria. Overall, the median age of the population was 53 years (range 18–91 years) and 59% were male. Except for the group with old patients, all groups had more males compared to females (Table 1). The young, middle-aged, and the elderly groups, respectively, had 34.6, 42.1, and 42.7% of female patients compared to 51.9% of females in the old group. Overall, the median time since diagnosis for the patients was 0.9 months (range <0.1–6.6 months) and was similar when evaluated by age group (Table 1). According to the Sokal risk score, overall, 377 patients (34.6%) were categorized as low risk, 408 (37.5%) as intermediate risk, and 197 (18.1%) as high risk; Sokal risk score could not be calculated for 107 patients (9.8%) due to missing information. More than 80% of patients (*n* = 900, 82.6%) were considered to be at low risk based on the EUTOS score and 8.6% (*n* = 94) at high risk, and information was missing for 8.7% (*n* = 95). In each of the age category, most of the patients were at low or intermediate risk based on the Sokal risk score and low risk based on the EUTOS risk score (Table 1). Based on the Sokal risk score, none of the patients in the old group were at low risk, while about half of the patients in the young (55.6%) and the middle age groups (41.1%) were at low risk. More than half of the patients in the elderly (53.0%) and in the old (69.2%) groups were at intermediate risk for Sokal score. Based on the EUTOS risk score, a slightly higher percentage of patients were at high risk in young age group (12.8%) compared to the other age categories. The percentages of eosinophils, basophils, and platelets were similar across the age groups, but the percentage of blasts was slightly higher, and the spleen size was comparatively bigger in the young patients than in the other age groups.

**Table 1:** Baseline demographics

Characteristics	Young (18–39 years) (*n* = 243)	Middle age (40–59 years) (*n* = 494)	Elderly (60–74 years) (*n* = 300)	Old (≥75 years) (*n* = 52)
Median age (range), years	32 (18–39)	50 (40–59)	66 (60–74)	78 (75–91)
Male/female (%)	159/84 (34.6%)	286/208 (42.1%)	172/128 (42.7%)	25/27 (51.9%)
Time since initial diagnosis of CML (months); median (range)	0.86 (0.07, 5.86)	0.92 (0.07, 6.61)	0.92 (0.03, 60.99)	0.86 (0.07, 6.02)
Sokal score, *n* (%)				
High risk	31 (12.8)	84 (17.0)	71 (23.7)	11 (21.2)
Intermediate risk	52 (21.4)	161 (32.6)	159 (53.0)	36 (69.2)
Low risk	135 (55.6)	203 (41.1)	39 (13.0)	–
Missing	25 (10.3)	46 (9.3)	31 (10.3)	5 (9.6)
EUTOS score, *n* (%)				
High risk	31 (12.8)	39 (7.9)	22 (7.3)	2 (3.8)
Low risk	190 (78.2)	412 (83.4)	252 (84.0)	46 (88.5)
Missing	22 (9.1)	43 (8.7)	26 (8.7)	4 (7.7)
Laboratory parameters				
Peripheral blasts %, mean ± SD (*n*)	2.05 ± 3.03 (230)	1.60 ± 2.33 (475)	1.49 ± 2.25 (283)	0.98 ± 1.04 (48)
Peripheral eosinophils %, mean ± SD (*n*)	2.88 ± 3.37 (234)	2.73 ± 2.64 (479)	2.85 ± 3.14 (289)	2.47 ± 2.37 (49)
Peripheral basophils %, mean ± SD (*n*)	4.02 ± 3.75 (234)	4.07 ± 3.83 (478)	4.08 ± 3.95 (292)	3.89 ± 3.72 (50)
Platelets (10^9^/L), mean ± SD (*n*)	446.47 ± 312.07 (240)	464.08 ± 352.63 (487)	463.28 ± 308.61 (297)	423.69 ± 233.39 (51)
Spleen size, cm	5.01 ± 6.19 (226)	3.22 ± 4.90 (461)	2.46 ± 4.13 (276)	1.53 ± 3.06 (49)
Previous CML therapy, *n* (%)				
Imatinib ≤1 month	17 (7)	32 (6.5)	16 (5.3)	2 (3.8)
Imatinib >1–2 months	13 (5.3)	33 (6.7)	24 (8.0)	1 (1.9)
Imatinib >2–3 months	16 (6.6)	20 (4)	10 (3.3)	4 (7.7)

*CML* chronic myeloid leukemia, *EUTOS* European Treatment Outcome Study

Of the 1089 patients enrolled, 881 (80.9%) completed 24 months of study treatment and 208 (19.1%) discontinued. Discontinuation rates were 16.9% in young patients, 16.2% in middle-aged patients, 22.3% in elderly, and 38.5% in old patients, which was the highest among the four age groups (Fig. 1). Across all age groups, the most common reason for discontinuation was AEs, accounting for 36.6, 61.2, 61.2, and 60% of all discontinuations in the young, middle-aged, elderly, and old patients, respectively. On the other hand, of the patients discontinued, 17.1, 8.1, 4.5%, and none discontinued due to disease progression or treatment failure in the young, middle-aged, elderly, and old age groups, respectively.

The overall median (range) duration of exposure was 722 days (5–821 days) and did not vary much across the four age groups studied (Table 2). The median dose intensity was around 600 mg/day across all groups. A total of 492 patients (45.2%) underwent dose change or interruptions. Dose reductions were comparatively less in old patients, while dose interruptions were more frequent in this group compared to the other groups (Table 2).

**Table 2:** Drug exposure

	Young (*n* = 243)	Middle age (*n* = 494)	Elderly (*n* = 300)	Old (*n* = 52)
Duration of exposure, days (median, range)	722.00 (46–821)	724 (5–793)	724 (1–798)	709 (4–780)
Time of treatment, days (median, range)	728 (46–960)	728 (5–888)	728 (1–817)	718 (4–780)
Average daily dose, mg/day, (mean ± SD)	583.1 ± 50.7	587.0 ± 46.4	577.6 ± 66.4	576.5 ± 64.9
Dose intensity, mg/day (median, range)	600 (257.8–620.5)	600 (144.4–689.0)	600 (173.9–625.7)	598.5 (300.0–600.0)
Dose reductions, *n* (%)	71 (29.2%)	146 (29.6%)	90 (30.0%)	14 (26.9%)
1 Dose reduction	32 (13.2)	76 (15.4)	46 (15.3)	6 (11.5)
2 Dose reductions	15 (6.2)	30 (6.1)	20 (6.7)	5 (9.6)
>2 Dose reductions	24 (9.9)	40 (8.1)	24 (8)	3 (5.8)
Dose interruptions, *n* (%)	84 (34.6%)	168 (34.0%)	113 (37.7%)	22 (42.3%)
1 Dose interruption	39 (16.1)	95 (19.2)	57 (19.0)	14 (26.9)
2 Dose interruptions	20 (8.2)	44 (8.9)	21 (7.0)	4 (7.7)
>2 Dose interruptions	25 (10.3)	29 (5.9)	35 (11.7)	4 (7.7)

### Molecular response rates

**Fig. 2:**
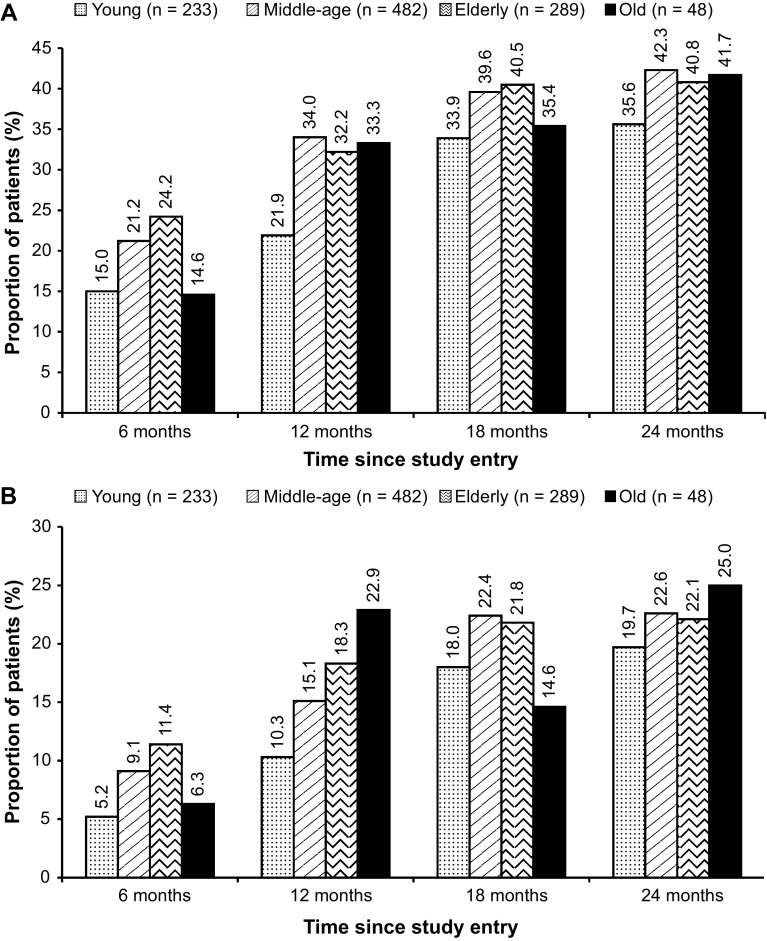
Molecular response rates in the molecular analysis population (*n* = 1052). **a** Rates of MR^4^ at 6, 12, 18, and 24 months by age group. *MR* ^4^ molecular response 4 (MR^4^; *BCR–ABL1* ^IS^ ≤0.01), *IS* international scale. **b** Rates of MR^4.5^ at 6, 12, 18, and 24 months by age group. *MR* ^4.5^ molecular response 4.5 (MR^4.5^; *BCR–ABL1* ^IS^ ≤0.0032%), *IS* international scale

Among the 1082 patients, 1052 were considered for the evaluation of the molecular response rates. Overall, the rate of MR^4^ at 18 months was 38.4% (95% CI, 35.5–41.3%; *n* = 404). At 18 months, the rates of MR^4^ were 33.9% (95% CI, 27.8–40.0%) in the young adult patients, 39.6% (95% CI, 35.3–44.0%) in the middle-aged patients, 40.5% (95% CI, 34.8–46.1%) in the elderly patients, and 35.4% (95% CI, 21.9–48.9%) in the old patients (Fig. 2a). The rates of MR^4.5^ at 18 months were 18% (95% CI, 13.1–23.0%), 22.4% (95% CI, 18.7–26.1%), 21.8% (95% CI, 17.0–26.6%), and 14.6% (95% CI, 4.6–24.6%) in the young adults, middle-aged, elderly, and old patients, respectively. The MR^4^ and MR^4.5^ rates at 6, 12, and 24 months are presented in Fig. 2. In the overall population, cumulative rates of MR^4^ and MR^4.5^ by 24 months were 55.2% (*n* = 581) and 38.6% (*n* = 406), respectively. By 24 months, the rates of MR^4^ among patients were 50.2% (95% CI, 43.8–56.6%; *n* = 117) in the young age group, 57.1% (95% CI, 52.6–61.5%; *n* = 275) in the middle-aged group, and 57.4% (95% CI, 51.7–63.1%; *n* = 166) and 47.9% (95% CI, 33.8–62.0%; *n* = 23) in the elderly and the old patients, respectively. The rates of MR^4.5^ in the young adults, middle-aged, elderly, and old patients were 35.6% (95% CI, 29.5–41.8%; *n* = 83), 39.4% (95% CI, 35.1–43.8%; *n* = 190), 39.8% (95% CI, 34.1–45.4%; *n* = 115), and 37.5% (95% CI, 23.8–51.2%; *n* = 18) by 24 months, respectively. The rates of MR^4^ based on the Sokal risk score are presented in Table 3. Similar rates of MR^4^ were seen across the age groups, except for the young adult patients with intermediate risk and old patients with high risk, which showed considerably lower rates of MR^4^; 14 of 49 patients (28.6%) in the young group with intermediate risk and 2 of 10 patients (20.0%) in the old group with high risk achieved MR^4^.

**Table 3:** Effect of Sokal risk on the MR^4^ by age group

MR^4^ by 24 months by Sokal risk group, %	Young (*n* = 233)	Middle age (*n* = 482)	Elderly (*n* = 289)	Old (*n* = 48)
Low	56.9	62.3	63.2	–
Intermediate	28.6	54.1	58.4	51.5
High	44.8	46.4	54.4	20.3

*MR* ^4^ molecular response 4 (*BCR–ABL1* ^IS^ ≤0.01%), *IS* international scale

### Overall survival and progression-free survival

Overall, for the patients included in the ITT set, the estimated OS at 12 months was 99.6% (95% CI, 99.0–99.9%) and at 24 months was 98.9% (95% CI, 98.0–99.4%). There were 18 deaths reported in this study, of which 14 occurred more than 28 days after the last dose of the study drug. The four deaths occurring within 28 days of the last dose of the study drug or the 24-month evaluation included death due to pulmonary embolism, aortic valve stenosis, thrombocytopenia, and pneumonia (one patient each). None of the four deaths were suspected to be related to the study drug. The 14 deaths occurring after 28 days of the last dose of the study drug or the 24-month evaluation included death due to secondary cancer (three patients), sepsis (two patients), unknown cause (two patients), progression of CML, pneumonia, cardiac failure, cerebral infarction, ischemic stroke, complications during a stem cell transplant, and suicide (one patient, each).

Overall, there were six progression events, three patients each on treatment progressed to AP and BC, though none of them died on the study. Of the six events, two were observed in the young group and four in the middle-aged group. By 24 months, the estimated rate of freedom from progression to AP/BC on treatment was 99.1% (95% CI, 97.7–99.7%) in middle-aged and 99.2% (95% CI, 96.6–99.8%) in young patients, while it was 100% in both the elderly and old patients.

### Adverse events

The overall safety results of the ENEST1st study are presented in Hochhaus et al. (2016a). The most common nonhematological AEs included rash, pruritus, headache, abdominal pain, fatigue, nausea, alopecia, and nasopharyngitis (Hochhaus et al. 2016a). Although the incidence of AEs was slightly different, no new specific safety signals were observed across the four age groups (Table 4). The most frequent nonhematological AEs were rash in young (21.4%) and the middle-aged (24.9%), and pruritus (18.3%) and nausea (19.2%) in elderly and old patients, respectively. Among hematological AEs, thrombocytopenia was the most frequent AE that was observed in young (14.8%), middle-aged (9.3%), and elderly patients (9.3%), while in old patients anemia (15.4%) was the more common hematological AE (Table 5). The common biochemical laboratory abnormalities included increase in bilirubin, increase in alanine aminotransferase (ALT), decrease in phosphate, increase in lipase, and arterial hypertension (Table 6). Among the age groups, arterial hypertension was less frequent in young patients (2.9%) compared to the other age groups (middle-aged, 6.3%; elderly, 7.3%; old, 9.6%); while increases in bilirubin, ALT, and aspartate aminotransferase (AST) were less frequent or absent in the old population. Frequency of grade 3 or 4 abnormalities was relatively less for nonhematological AEs compared to the hematological AEs. Of the total incidence of thrombocytopenia and neutropenia, more than 50% were of grade 3 or 4 except in the old patients, in whom it was only 1.9%. Overall, the incidence of grade 3 or 4 AEs was less frequent in old patients.

**Table 4:** Most frequent all grades (≥10% in any group) nonhematological adverse events (AEs)

AEs	Young (*n* = 243), *n* (%)		Middle age (*n* = 494), *n* (%)		Elderly (*n* = 300), *n* (%)		Old (*n* = 52), *n* (%)	
	All grades	Grade 3 or 4	All grades	Grade 3 or 4	All grades	Grade 3 or 4	All grades	Grade 3 or 4
Abdominal pain	33 (13.6)	1 (0.4)	74 (15.0)	5 (1.0)	45 (15.0)	2 (0.7)	8 (15.4)	–
Rash	52 (21.4)	1 (0.4)	123 (24.9)	1 (0.2)	52 (17.3)	1 (0.3)	6 (11.5)	–
Pruritus	33 (13.6)	1 (0.4)	83 (16.8)	1 (0.2)	55 (18.3)	1 (0.3)	9 (17.3)	–
Fatigue	31 (12.8)	1 (0.4)	76 (15.4)	4 (0.8)	41 (13.7)	1 (0.3)	3 (5.8)	–
Headache	51 (21.0)	5 (2.1)	86 (17.4)	3 (0.6)	25 (8.3)	–	4 (7.7)	–
Nausea	19 (7.8)	2 (0.8)	59 (11.9)	–	35 (11.7)	3 (1.0)	10 (19.2)	–
Nasopharyngitis	36 (14.8)	–	44 (8.9)	–	31 (10.3)	–	2 (3.8)	–
Alopecia	25 (10.3)	1 (0.4)	59 (11.9)	–	24 (8.0)	–	7 (13.5)	–
Dry skin	17 (7.0)	–	43 (8.7)	–	30 (10.0)	–	3 (5.8)	–
Myalgia	26 (10.7)	1 (0.4)	52 (10.5)	–	19 (6.3)	1 (0.3)	2 (3.8)	–
Muscle spasm	13 (5.3)	–	48 (9.7)	–	30 (10.0)	–	2 (3.8)	–
Diarrhea	16 (6.6)	-	39 (7.9)	1 (0.2)	35 (11.7)	1 (0.3)	4 (7.7)	–

**Table 5:** Most frequent hematologic adverse events (AEs) (≥5% in any group) of interest

AEs	Young, *n* (%)		Middle age, *n* (%)		Elderly, *n* (%)		Old, *n* (%)	
	All grades	Grade 3–4	All grades	Grade 3–4	All grades	Grade 3–4	All grades	Grade 3–4
Thrombocytopenia	36 (14.8)	20 (8.2)	46 (9.3)	28 (5.6)	28 (9.3)	17 (5.7)	3 (5.8)	1 (1.9)
Anemia	13 (5.3)	4 (1.6)	28 (5.7)	5 (1.0)	18 (6.0)	7 (2.3)	8 (15.4)	1 (1.9)
Neutropenia	14 (5.8)	12 (4.9)	20 (4.0)	11 (2.2)	7 (2.3)	6 (2.0)	2 (3.8)	1 (1.9)
Leukopenia	7 (2.9)	1 (0.4)	13 (2.6)	4 (0.8)	5 (1.7)	3 (1.0)	–	–
Lymphopenia	2 (0.8)	–	1 (0.2)	–	1 (0.3)	–	–	–

**Table 6:** Most frequent (≥5% in any group) laboratory abnormalities

Laboratory abnormalities	Young, *n* (%)		Middle age, *n* (%)		Elderly, *n* (%)		Old, *n* (%)	
	All grade	Grade 3 or 4	All grade	Grade 3 or 4	All grade	Grade 3 or 4	All grade	Grade 3 or 4
Total bilirubin ↑	25 (10.3)	5 (2.1)	36 (7.3)	5 (1.0)	17 (5.7)	4 (1.3)	2 (3.8)	
ALT ↑	27 (11.1)	4 (1.6)	49 (9.9)	9 (1.8)	9 (3.0)	2 (0.7)	1 (1.9)	1 (1.9)
AST ↑	14 (5.8)	1 (0.4)	26 (5.3)	4 (0.8)	11 (3.7)	1 (0.3)	–	
Phosphate ↓	13 (5.3)	6 (2.5)	48 (9.7)	15 (3.0)	15 (5.0)	3 (1.0)	1 (1.9)	
Lipase ↑	7 (2.9)	3 (1.2)	37 (7.5)	16 (3.2)	30 (10)	18 (6.0)	3 (5.8)	1 (1.9)
Hypertension	7 (2.9)		31 (6.3)	4 (0.8)	22 (7.3)	6 (2.0)	5 (9.6)	2 (3.8)

*ALT* alanine aminotransferase, *AST* aspartate aminotransferase

The cardiovascular AEs by age group are presented in Table 7. By Fisher’s exact test, there was a significant difference (*P ≤*0.0001) in the incidence of cardiovascular events (CVEs) overall, across the age groups, with very low incidence in the young patients compared to the other age groups (Table 7). Among the CVEs, the incidence of ischemic heart disease was significantly different across age groups (*P* <0.0002), while peripheral arterial occlusive disease and ischemic cerebrovascular event did not differ significantly across age groups. It was not mandatory to monitor the lipid profile and glucose routinely as per the protocol. However, AEs related to hypercholesterolemia (3.0%), hyperglycemia (3.3%), and diabetes mellitus (1.2%) were reported earlier for the overall population (Hochhaus et al. 2016a).

**Table 7:** Cardiovascular adverse events (AEs) by age group

Cardiovascular AEs	Young (*n* = 243)	Adult (*n* = 494)	Elderly (*n* = 300)	Old (*n* = 52)	*P* value by Fisher’s exact test*
Cardiovascular events	2 (0.8%)	26 (5.3%)	30 (10%)	7 (13.5%)	<0.0001
Ischemic heart disease	1 (0.4%)	14 (2.8%)	17 (5.7%)	5 (9.6%)	0.0002
Peripheral arterial occlusive disease	1 (0.4%)	9 (1.8%)	9 (3.0%)	1 (1.9%)	0.12
Ischemic cerebrovascular event	0	4 (0.8%)	4 (1.3%)	1 (1.9%)	0.19

*P values provided are nominal, post hoc, and for descriptive purpose only; no multiplicity adjustments were made

## Discussion

The ENEST1st study evaluated deep molecular response with nilotinib in newly diagnosed patients with CML (Hochhaus et al. 2016a). This subanalysis of the ENEST1st study was conducted to assess the impact of age on deep molecular response and AEs with frontline nilotinib. The ENEST1st study had shown that among the patients analyzed for molecular response, 38.4% achieved MR^4^ at 18 months and 55.2% achieved MR^4^ by 24 months. When categorized into 4 age groups comprising young, middle age, elderly, and old patients, molecular response rates across the groups were consistent with the overall population. To the best of our knowledge, this subanalysis is the first to compare the safety and efficacy of frontline nilotinib across the four different age groups.

In the earlier studies with busulfan, hydroxyurea, and allogeneic stem cell transplantation, older age was considered to negatively impact the response and survival outcomes (Silver et al. 1999; Berger et al. 2003) and indicated poor prognosis in patients with CML (Kantarjian et al. 1987). However, later studies evaluating the impact of age on response rates with imatinib have been conflicting. In a study by Rosti et al., in patients with CML in late CP, lower rates of complete hematologic response (CHR) and complete cytogenetic response (CCyR) were observed in patients >65 years compared to younger patients, though OS was the same (Rosti et al. 2007). Gugliotta et al. in 2011, however, suggested no impact of age on the response rates upon treatment with imatinib (Gugliotta et al. 2011). However, it should be noted that there was a slight difference in the disease stage of the patients in the two studies. The first study included patients in late chronic phase who were resistant to interferon alpha, treated with imatinib, while the second study included patients in early chronic phase treated with frontline imatinib. Earlier in a large study of more than 700 patients at the MD Anderson center, no differences in the CCyR and OS were observed between patients over 60 years and younger patients (Cortes et al. 2003).

A few studies have evaluated the impact of age on the safety and efficacy of second-generation TKIs in elderly. In the phase 2 study, in imatinib-resistant/intolerant patients who were treated with nilotinib, more patients <65 years of age achieved major cytogenetic response (MCyR; 63%) and CCyR (44%), compared with patients ≥65 years (MCyR, 48%; CCyR, 38%) (Lipton et al. 2008). The estimated OS rates at 12 months were higher for patients <65 years (97%) compared with patients ≥65 years (91%) (Lipton et al. 2008; le Coutre et al. 2009). However, in the ENACT study, which evaluated the response rates for patients ≥60 years, rate of CHR was comparable to that of the overall population (le Coutre et al. 2009). In the pivotal phase 3 ENESTnd study, MMR rates by 24 months for patients aged ≥65 years and those <65 years were similar for those treated with 300 mg but was slightly lower for the older patients (61%) on 400 mg of nilotinib bid, compared to younger patients (67%) (Larson et al. 2011). A subanalysis in the pivotal DASISION trial, which compared frontline imatinib with dasatinib, was one of the very few trials that compared the response rates in three different age groups comprising patients <46 years, patients aged between 46 years and 55 years, and those aged >65 years (Khoury et al. 2010). The response rates did not vary substantially with CCyR rates in the three groups ranging between 78 and 88% and MMR rates from 45 to 50%.

A number of studies have demonstrated that deep molecular response with TKIs resulted in better outcomes in patients with CML (Falchi et al. 2013). In the ENESTcmr study, patients who could not achieve molecular response with imatinib could achieve it after switching to nilotinib; while in the ENESTnd study more patients achieved MR^4^ and MR^4.5^ with nilotinib compared to imatinib (Hughes et al. 2014; Hochhaus et al. 2016b). The ENEST1st study conducted in a large patient population of more than 1000 patients further confirmed the efficacy of frontline nilotinib with a better response than in the ENESTnd study, in which 39 and 25% achieved MR^4^ and MR^4.5^ by 24 months, compared to 56 and 38% in the ENEST1st study, respectively (Hochhaus et al. 2016a).

Most studies, including the ENESTnd that evaluated the impact of age, have categorized patients broadly into elderly patients who were older than 60 or 65 years and those who were younger. The classification made in this analysis, with subgroups ranging from 15 to 20 years each, would enable us to identify any differences, which may be lost due to a broader classification. The current study did not show any major difference between the age groups analyzed, though comparatively weaker responses were seen for younger patients compared to the older patients. The younger patients in particular had a higher percentage of blasts and spleen size compared to the older patients as has also been reported in other studies (Pemmaraju et al. 2012; Kalmanti et al. 2014; Castagnetti et al. 2015). Somewhat similar approach has earlier been seen with the CML IV study with >1500 patients and the GIMEMA CML working group studies with >2500 patients, which were large studies and also evaluated the impact of age in patients with CML treated with TKIs. In these studies (Kalmanti et al. 2014; Castagnetti et al. 2015), in which the patients were classified into 3 to 4 categories of age, as in the current study, it was seen that younger patients with CML typically present with a more expanded disease (Pemmaraju et al. 2012; Kalmanti et al. 2014; Castagnetti et al. 2015) and a higher incidence of hematologic toxicity as also seen here (Pemmaraju et al. 2012). In a study by Kalmanti et al., poor prognostic indicators in younger patients did not seem to affect their response to frontline imatinib (Kalmanti et al. 2014). However, according to Pemmaraju et al. young adults showed comparatively lower response rates to frontline TKIs compared to older patients, though transformation-free survival, and the OS remained similar (Pemmaraju et al. 2012). Further analysis in this age group might be needed. Since the current study enrolled newly diagnosed patients according to protocol inclusion and exclusion criteria, the elderly and the old patients could have been healthier; hence, better efficacy and tolerability to some drug- or disease-related AEs were seen compared to young adults. The responses could also reflect a possible selection bias of the investigators in recruiting younger patients into the clinical trials compared to population-based registries as is also reported by other authors (Rohrbacher et al. 2009).

Recently, the therapeutic landscape of CML is moving toward the goal of achieving TFR, and one of the major criteria for patients to attempt to discontinue treatment is a sustained molecular response (Hughes and Ross 2016). Many studies investigating the predictors of successful TFR have also evaluated age as a potential predictor (Lee et al. 2016; Etienne et al. 2017). The ENEST1st study evaluated deep molecular response with frontline nilotinib, which potentially indicates that the population may be eligible to attempt TFR. The impact on age, if any, on attaining deep molecular response may indicate potential differences in the age of the population, which can attempt TFR. However, since no significant differences were observed in the response rates with the different age groups, an impact of age on the patients attempting TFR or on the outcome of TFR may not be likely. This is further indicated in the studies, in which age was not found to be a predictor for outcomes of TFR (Lee et al. 2016).

Overall, safety was consistent with the known profile of nilotinib. The overall safety signals for the ENEST1st study were similar to those of the ENESTnd, though at a lower frequency than ENESTnd suggesting better management of the disease in this study (Hochhaus et al. 2016a). Although the distribution of some of the AEs differed across age groups, safety signals specific for a particular age group could not be identified except for CVEs, which were significantly less for the young patients. These data highlight the need for appropriate monitoring for relevant risk factors in all patients receiving nilotinib therapy with immediate appropriate intervention when needed, especially if a CVE occurs. (Rea et al. 2015; Castagnetti et al. 2016).

Even though the study enrolled a large patient population with sufficient number of patients in each group, the subanalysis was not designed to formally test the difference across the subgroups. In the ENEST1st study, monitoring of glucose and lipid was not mandatory and the overall frequency of these AEs and also their differences with age, if any, could not be ascertained and were probably underestimated. The current classification used in the study does not conform to any standard classification of age, e.g., the US census bureau or World Health Organization, and was done to introduce additional categories, though arbitrarily, to identify differences in population if any.

The ENEST1st study had shown high molecular response rates with approximately 55.2% of the patients achieving MR^4^ by 24 months. This subanalysis of the ENEST1st study showed that age had minimal impact on the deep molecular response rates associated with nilotinib therapy in newly diagnosed patients with CML and eventually on the eligibility of the patients to attempt TFR. This together with almost 100% freedom from progression by 24 months in any of the age groups further demonstrated the efficacy of frontline nilotinib. Although the main causes of discontinuation were similar across the young, middle-aged, elderly, and old patients, the distribution varied slightly across the age groups. Understanding the variations in disease characteristics and AEs with TKI therapy with respect to patient age may help improve CML therapy. Especially in older patients with a higher proportion of comorbidities, a more flexible dosing scheme may be warranted to increase tolerability while maintaining the deep molecular responses.
